# Analysis of blood screening strategies and their efficacy among voluntary blood donors in a region of East China

**DOI:** 10.1371/journal.pone.0331027

**Published:** 2025-08-21

**Authors:** Yiming Jin, Rong Lu, Mingyuan Wang, Zihao Xu, Zhen Liu, Shuhong Xie, Yu Zhang

**Affiliations:** 1 Department of Blood Screening Test, Suzhou Blood Center, Suzhou, China; 2 Division of Transfusion Medicine, Suzhou Blood Center, Suzhou, China; 3 Department of Blood Screening Test, Suzhou Municipal Hospital, Suzhou, China; Kwame Nkrumah University of Science and Technology, GHANA

## Abstract

**Objective:**

In this study, we aimed to analyze the blood screening detection strategies employed for voluntary blood donation in a specific region of East China and evaluate the efficacy of the blood safety detection system.

**Donors and Methods:**

A total of 539,117 whole blood samples were collected from voluntary blood donors between January 2018 and July 2021, as well as in 2023 and 2024. The samples were screened for hepatitis B surface antigen (HBsAg), hepatitis C virus (HCV) antibodies, human immunodeficiency virus antibodies/antigen (HIV Ab/Ag), and *Treponema pallidum* (TP) antibodies using enzyme-linked immunosorbent assay (ELISA). Alanine aminotransferase (ALT) levels were measured using a rapid method. Chemiluminescence immunoassay technology was used to detect five hepatitis B virus (HBV) markers. Polymerase chain reaction was employed to detect HBV DNA, HCV RNA, and HIV RNA. The reactivity rates of each marker were analyzed.

**Results:**

The overall positivity rate for blood testing among donors in this region was 0.76% (4,078/539,117). The positivity rates for the individual markers were as follows: anti-TP (0.20%)> HBsAg (0.18%)> ALT (0.13%)> anti-HCV (0.085%)> nucleic acid testing (0.080%)> HIV antigen/anti-HIV (0.079%). No significant differences were observed (P > 0.05). Before 2023, the positivity rates for ALT and HBsAg exhibited occasional fluctuations, followed by a significant decline. Conversely, in 2024, a slight upward trend in the HIV positivity rate was noted.

**Conclusion:**

The current multitiered blood screening and detection strategy in this region exhibits complementary advantages, ensuring effective blood safety. However, the observed slight upward trend in the HIV positivity rate among voluntary blood donors highlights the necessity for enhanced pre-donation counseling and risk assessment for key populations.

## Introduction

Blood screening is a critical step to ensure the safety of clinical blood use. During blood collection from voluntary donors, obtaining specimens that meet all necessary testing criteria is essential. These specimens undergo rigorous evaluation, encompassing blood type identification and serological and nucleic acid testing (NAT) for transfusion-transmissible infectious disease markers, in addition to alanine aminotransferase (ALT) testing [1]. Transfusion-related infectious disease markers cover the infection markers of human immunodeficiency virus (HIV), hepatitis B virus (HBV), hepatitis C virus (HCV), and *Treponema pallidum* (TP), and blood can only be released after all the testing items are qualified. Currently, blood stations in China adhere to unified screening standards. However, the test results may vary owing to differences in reagent usage and study populations. In addition, some local regions have implemented donor reentry programs to retain donors. These programs reevaluate the status of certain voluntary donors who were initially disqualified during screening, thereby enabling those who have lost their eligibility because of false-positive reactions to regain their right to donate. This helps to reduce the loss of donors. In the present study, we conducted a retrospective analysis of voluntary blood donors from 2018 to 2024, evaluating blood screening strategies and their effectiveness as implemented by blood stations in the region.

## Materials and methods

### Blood samples information

From January 2018 to July 2021, as well as throughout 2023 and 2024, a total of 539,117 blood screening specimens were collected from voluntary donors at Suzhou Blood Center in Jiangsu Province, Eastern China. This study complied with China’s blood donation policies, which restrict donors to adults aged 18–60 years. Due to significant disruptions in blood collection during the COVID-19 pandemic, data from August 2021 to December 2022 were excluded from analysis. This exclusion was necessary because reduced donor turnout and operational challenges during this period resulted in limited population representation and compromised data quality. We analyzed retrospective data from fully anonymized blood donor screening records (January 2018-December 2024). Prior to analysis, Suzhou Blood Center’s data custodians removed all personal identifiers (including names, contact information, and national ID numbers), retaining only laboratory-assigned codes linked to test results (e.g., ELISA S/CO ratios, NAT Ct values). All study data were accessed from the Suzhou Blood Center’s secure donor database between January 2 and February 28, 2025. The research team performed daily data exports during this period, with final validation completed on Feb 28 2025 prior to analysis.

### Screening procedures

**Fig 1:**
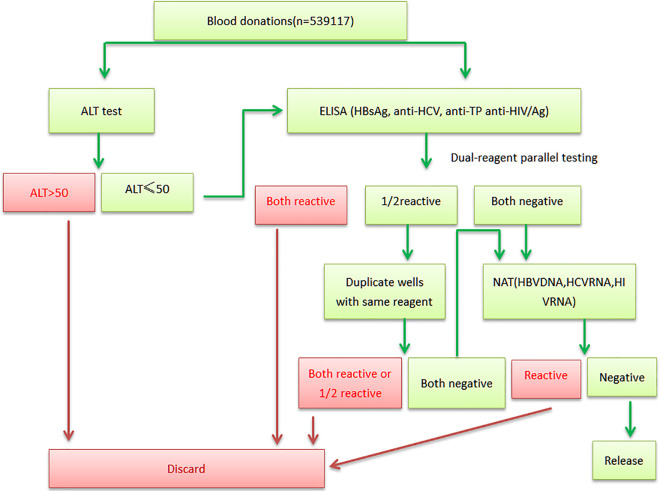
Blood screening workflow for voluntary donors.

All samples were analyzed strictly following the guidelines of *The Technical Operating Procedures for Blood Stations (2019 Edition).* The screening algorithm is illustrated in Fig 1. Briefly, ALT activity is quantitatively determined using the kinetic method, following standardized clinical laboratory protocols. Enzyme-linked immunosorbent assay (ELISA) is performed using reagents from two distinct manufacturers for hepatitis B surface antigen (HBsAg), hepatitis C virus antibody (Anti-HCV), Treponema pallidum antibody (Anti-TP), and human immunodeficiency virus antigen/antibody (HIV Ag/Ab). If a positive result is obtained with either reagent, duplicate-well retesting must be conducted using the same reagent. Samples demonstrating positivity with both reagents are immediately classified as reactive. ELISA (HBsAg, Anti-HCV, HIV Ag/Ab) negative specimens proceed to NAT for further analysis. To ensure transfusion safety, only specimens with all final results confirmed negative are released to hospitals. For donor reentry, all specimens shall complete the full retesting procedure specified in Fig 1, with requalification permitted only after negative confirmation. The characteristics of the commercial kits used were summarized in Table 1.

**Table 1:** Characteristics of commercial kits used in this study.

Test	Assay	Cutoff criteria used	LOD
ALT	kinetic method	Positive: ALT > 50U/mL; Negative:ALT ≤ 50 U/mL	/
HBsAg	Sandwich assay	**Kehua/ Xinchuang:** Positive: S/CO ≥ 1 Gray zone: 0.8 ≤ S/CO < 1 Negative: S/CO < 0.8,	≥0.1 IU/mL
Anti-HCV	Indirect method (Xin chuang) sandwich assay (Wantai)	**Xinchuang:** Positive: S/CO ≥ 1; Gray zone: 0.8 ≤ S/CO < 1 Negative: S/CO < 0.8, **Wantai:** Positive: S/CO ≥ 1 Gray zone: 0.8 ≤ S/CO < 1 Negative: S/CO < 0.8	Xinchuang: ≥ 0.3NCU/mL Wantai: ≥ 0.02NCU/mL
Anti-TP	Sandwich assay	**Xinchuang:** Positive: S/CO ≥ 1 Gray zone: 0.8 ≤ S/CO < 1 Negative: S/CO < 0.8 **Wantai:** Positive: S/CO ≥ 1 Gray zone: 0.8 ≤ S/CO < 1 Negative: S/CO < 0.8	Xinchuang: ≥ 3mIU/mL Wantai: ≥ 1.5mIU/mL
HIV Ag/Ab	3G (Xinchuang) 4G (Wantai)	**Xinchuang:** Positive: S/CO ≥ 1 Gray zone: 0.8 ≤ S/CO < 1 Negative: S/CO < 0.8, **Wantai**: Positive: S/CO ≥ 1 Gray zone: 0.8 ≤ S/CO < 1 Negative: S/CO < 0.8	Xinchuang: Ab ≥ 0.2 NCU/mL Wantai: Ab ≥ 0.2 NCU/mL, p24 Ag ≥ 1.5U/mL
HBVDNA/HCVRNA/HIVRNA	Realtime-PCR(Roche)	Automatically interpreted by the software.	HBVDNA≥2.3 IU/mL; HCVRNA≥6.8 IU/mL; HIVRNA:HIV-1M ≥ 46.2 IU/mL, HIV-2 ≥ 7.9 IU/mL
HBsAg	Chemiluminescence immunoassay(Roche)	Quantitative	≥0.05 IU/mL
Anti-HBs		Quantitative	≥2 mIU/mL
HBeAg		Quantitative	≥0.1PEI U/ml
Anti-HBe		Positive: COI ≥ 1,	/
Anti-HBc		Positive COI ≥ 1	/

S/CO: Sample OD/Cutoff value; LOD: Limit of detection; 3G/4G: Third-/Fourth-generation assay, COI: Cut-off Index

### Serological testing

Two types of enzyme-linked immunosorbent assay (ELISA) reagents were employed for detecting HBsAg (Kehua Kit, Shanghai Kehua Bio-engineering Co, China; Xinchuang Kit, InTec ADVANCED Diagnostic, China), anti-HCV antibodies (Wantai Diagnostic Kit, Beijing Wantai Biological Pharmacy Enterprise Co, China; Xinchuang Kit, InTec ADVANCED Diagnostic, China), HIV antigen/anti-HIV (Wantai Diagnostic Kit, Beijing Wantai Biological Pharmacy Enterprise Co, China; Xinchuang Kit, InTec ADVANCED Diagnostic, China), and anti-TP (Wantai Diagnostic Kit, Beijing Wantai Biological Pharmacy Enterprise Co, China; Xinchuang Kit, InTec ADVANCED Diagnostic, China) on the Fame24/30 fully automatically immunoassay analyzer (Hamilton, Switzerland). ALT levels were quantified using a rapid enzymatic method with the Au680 analyzer (Beckman Coulter, Brea, CA, USA).

### Nucleic acid testing and chemiluminescence detection

**All ELISA-negative specimens** were subjected to NAT using the Roche Cobas S201 system (Roche Molecular Systems, Inc. USA), and the results were automatically interpreted using the software. Additionally, from June to July 2021, 88 HBsAg ELISA single-reagent-positive specimens were tested for HBV DNA using NAT. An e411 electrochemiluminescence analyzer (Roche Molecular Systems, Inc. USA) was used to quantitatively detect five hepatitis B infection markers: HBsAg, hepatitis B surface antibody (HBsAb), hepatitis B e antigen (HBeAg), hepatitis B e antibody (HBeAb), and hepatitis B core antibody (HBcAb).

### Blood donor reentry

A cohorting strategy was implemented for 58 blood donors between January and July 2021 based on the results of ELISA and NAT. Donors with negative ELISA and negative NAT results were included in the cohort, whereas those with positive results in ELISA or NAT were excluded.

### Statistical analysis

Statistical analyses were performed using SPSS software, version 19 (IBM Corp., Armonk, NY, USA). Differences were evaluated using the chi-squared test, with a P-value of less than 0.05 indicating statistical significance.

### Ethics approval and informed consent

This study was conducted in accordance with the Declaration of Helsinki and approved by the Medical Ethics Committee of the Suzhou Blood Center (Approval number: 202406; November 1, 2024). For prospective components (e.g., donor reentry program): Written informed consent was obtained from all returning donors, which included authorization for the use of their screening data for research. For retrospective data analysis (2018–2024), the ethics committee waived the requirement for informed consent because the data were fully anonymized (with all personal identifiers removed) and aggregated for public health surveillance purposes.

## Results

**Fig 2:**
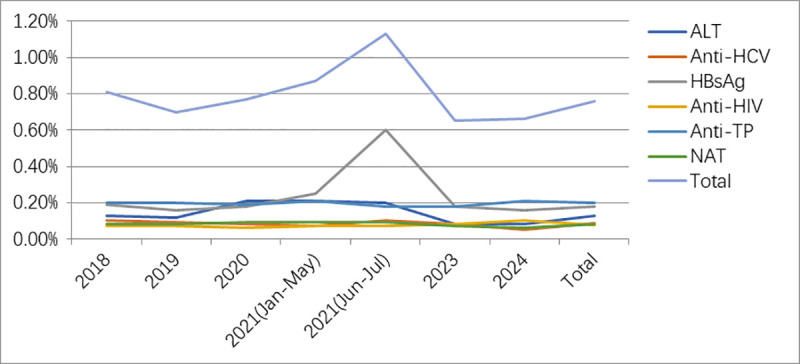
Trend chart of positivity rates for blood screening items from 2018-2024.

A comparison of the positivity rates for each item at different periods from 2018 to 2024 is shown in Table 2 and Fig 2.

**Table 2:** Comparison of the positivity rate from 2018-2024.

Year	2018	2019	2020	2021(Jan-May)	2021(Jun-Jul)	2023	2024	Total
N	93989	93659	94039	40791	17443	101185	98011	539117
ALT	0.13%	0.12%	0.21%	0.21%	0.20%	0.08%	0.08%	0.13%
Anti-HCV	0.10%	0.09%	0.08%	0.07%	0.10%	0.08%	0.05%	0.085%
HBsAg	0.19%	0.16%	0.18%	0.25%	0.60%	0.18%	0.16%	0.18%
Anti-HIV	0.07%	0.07%	0.06%	0.07%	0.07%	0.08%	0.10%	0.079%
Anti-TP	0.20%	0.20%	0.19%	0.21%	0.18%	0.18%	0.21%	0.20%
NAT	0.08%	0.08%	0.09%	0.09%	0.09%	0.07%	0.06%	0.08%
Total	0.81%	0.70%	0.77%	0.87%	1.13%	0.65%	0.66%	0.76%

Note: The positive detection rates for each item across different years were not statistically significant: χ^2^ < 0.01, p > 0.05

Demographic characteristics and reactivity rates of voluntary blood donors (2018–2024) are illustrated in Table 3.

**Table 3:** Demographic characteristics and reactivity rates of voluntary blood donors (2018-2024).

Category	Subcategory	Jan 2018-Jul 2021	2023	2024	Total	Reactive rate %	χ^2^	P-value
**Age Distribution**	18 ≤ Age < 25 years	91,727	20,820	16,893	129,440	0.07%	183.117	p = 0.000
	25 ≤ Age < 35 years	122,260	37,100	33,505	192,865	0.21%		
	35 ≤ Age < 45 years	80,455	29,710	31,490	141,655	0.28%		
	45 ≤ Age ≤ 55 years	40,624	12,919	13,687	67,230	0.14%		
	55 < Age ≤ 60 years	4,855	636	1,463	7,927	0.06%		
**Gender Distribution**	Male	231,066	74,382	72,096	377,544	0.45%	54.520	p = 0.000
	Female	108,855	26,803	25,915	161,573	0.31%		
**Education Level**	Primary School	/	/	/	/	/	/	/
	Junior High School	3,071	14,662	14,611	32,344	0.15%	17.914	p = 0.003
		Senior High School	5,070	15,094	14,463	34,627	0.15%	
		College (3-year)	1,959	14,773	15,249	31,981	0.12%	
		Undergraduate (4-year)	1,285	13,946	14,127	29,358	0.12%	
		Postgraduate	217	4,925	5,541	10,683	0.05%	
		Other/Unspecified	328,319	37,785	34,020	400,124	0.17%	
**Donation Frequency**	First-time Donors	177,569	53,564	50,216	281,349	0.48%	140.172	p = 0.000
	Repeat Donors	162,352	47,621	47,795	257,768	0.28%		
**Total Donors**		339,921	101,185	98,011	539,117	0.76%	/	/

The ELISA results of 88 HBsAg-positive samples (single-reagent testing) collected between June and July 2021 are presented in Table 4.

**Table 4:** Comparison of the results of 88 HBsAg single reagent positive specimens.

Reagent1(ELISA)		Reagent2 (ELISA)		HBVDNA		HBV (CLIA)				
S/CO	N	S/CO	N	Pos	Neg	HBsAg	HBsAb	HBeAg	HBeAb	HBcAb
>2.0	3	>2.0	11	0	14	0	2	0	0	1
>1.0	0	>1.0	13	0	13	0	1	0	0	0
<1.0	4	<1.0	57	0	61	0	0	0	0	0
Total	7		81	0	88	0	3	0	0	1

Note: S/CO: sample OD/Cutoff value; N: number; Pos: positive; Neg: negative

Results of 58 blood donor returns from January to July 2021 are summarized in Table 5.

**Table 5:** 58 cases of reentry blood donors.

HBsAg		Anti-HCV		Anti-HIV		Anti-TP		Total	
N	S	N	S	N	S	N	S	N	S
24	13	15	15	6	6	13	13	58	47

Note: N: number of total returns; S: number of successful returns

## Discussion

We comprehensively analyzed blood screening outcomes in 539,117 voluntary blood donors. As shown in Table 2, the overall ALT disqualification rate was 0.13%. Notably, the ALT positivity rate displayed marked fluctuations between January 2020 and July 2021, initially increasing from 0.12% to 0.21% before declining to 0.08%. However, these variations were not statistically significant (P > 0.05). Concurrently, the laboratory observed a substantial increase in lipid samples, suggesting a potential temporal association with the transient elevation in ALT positivity during this period. ALT, serving as a nonspecific marker for hepatitis screening, is notably susceptible to influence from various physiological conditions and nonhepatic pathological factors, which may lead to false-positive results in donor screening [1–2]. Internationally, the widespread implementation of advanced anti-HCV tests has led many countries (including those in Europe, North America, and Japan) to either eliminate mandatory ALT testing or increase its threshold values, due to its declining contribution to detect blood-borne pathogens (HBV, HCV, and HIV) [3–5].While ALT testing is still required for blood donation screening in China, authorities are now conducting a thorough reassessment of ALT cutoff levels [6–8]. The Chinese researcher Chang, through multicenter collaboration with provincial blood centers, demonstrated that adopting sex-specific ALT thresholds (≤100 U/L for males and ≤80 U/L for females) significantly increases donor eligibility without compromising blood safety, while substantially reducing blood discard rates [9]*.* By analyzing the demographic and physiological characteristics of blood donors with elevated ALT levels, Zhang X demonstrated that a scientifically calibrated adjustment of the ALT threshold could broaden the donor eligibility pool, mitigate blood supply shortages, and better align with advancements in modern testing methodologies [10]. These research findings can provide references for the optimization of the ALT threshold. However, in this study, we did not perform testing for blood-borne disease markers (HBV, HCV, and HIV) in ALT-disqualified donors, preventing a direct assessment of the association between elevated ALT levels and transfusion-transmissible infections. Future research should incorporate nucleic acid testing and serological assays to further analyze the correlation between ALT abnormalities and transfusion-transmitted infections, enabling a more evidence-based assessment of ALT screening’s clinical utility.

In our study, 0.08% (428/535,484) of ELISA-negative specimens showed NAT positivity, including 3 HIV RNA-positive cases (1 confirmed), 2 HCV RNA-positive cases (both negative confirmed), and HBV DNA-positive cases that were not followed up. The implementation of the national NAT mandatory screening policy in 2016 was a significant advancement in China’s blood safety management. The NAT screening yield in this region was 0.08% (428/535,484), demonstrating comparable detection rates to Xuzhou (0.1%) [11] and Wuxi (0.1%) [12], while being significantly higher than Xuchang [13] (0.03%) and Weifang (0.04%) [14] yet markedly lower than Guangdong (0.62%) [15] and Liangshan Prefecture (0.21%) [16]*.* The observed regional variations in NAT yield rates may be attributed to multiple technical and operational factors, including testing protocols, reagent manufacturers, laboratory conditions, and the proficiency of personnel. And these uniformly low residual NAT positivity rates reflect the effectiveness of China’s blood safety strategy, where initial ELISA screening eliminates the majority of seropositive donations prior to NAT testing. For blood borne viruses including HIV, HBV, and HCV, NAT exhibits enhanced sensitivity in identifying window-period infections and low-level viremia in donor specimens, providing critical complementation to the diagnostic gaps inherent in ELISA-based serological screening.

Regarding the serological monitoring of infectious disease markers, the current dual-reagent ELISA parallel testing system in China has exhibited remarkable stability [17–18]. Generally, the anti-HCV positivity rate remained consistent at 0.085%, whereas the HIV Ag/Ab positivity rate stabilized at 0.07%. The anti-TP positivity rate was consistently recorded at 0.20%. The enduring stability of these metrics suggests remarkable consistency in the demographic composition of the voluntary blood donor cohort, with a relatively stable proportion of regular donors.

In Table 2, the data demonstrate a sustained decline in the anti-HCV positivity rate, dropping from 0.10% in 2018 to 0.05% in 2024, though a transient rebound to 0.10% occurred during June-July 2021. This temporary increase may be attributed to pandemic-related testing delays or localized outbreaks. The significant decrease in anti-HCV positivity rate to 0.05% in 2024 suggests that recent HCV prevention and control measures have demonstrated effectiveness.

While the anti-HIV positivity rate remained stable at a low level (0.06%–0.08%), indicating a prevalence comparable to regions like Shaoxing (0.07%) [19] and Yantai (0.06%) [20]. This rate was significantly lower than high-prevalence areas such as Beijing (0.46%) [21] and Shijiazhuang (0.19%) [22], yet remained moderately higher than cities with exceptionally low rates including Guangzhou (0.03%) [23] and Nanjing (0.01%) [24]. These regional disparities are likely indicative of differences in population risk profiles, the effectiveness of local prevention programs, and patterns of urban mobility. Notably, the anti-HIV preliminary screening positivity rate showed a modest increase to 0.10% in 2024. While this upward trend remains limited in magnitude, it warrants careful monitoring. Longitudinal data from subsequent years (2025–2026) will be essential to determine whether this represents a sustained epidemiological shift. Furthermore, as a critical infectious disease indicator for targeted prevention and control, it is advisable to strengthen pre-donation counseling and risk assessment protocols for high-risk populations.

Data analysis revealed that from 2018 to 2024, the HBsAg positivity among blood donors remained consistently low, demonstrating remarkable stability within the range of 0.16% to 0.25%. In China, the general population has an HBsAg positivity rate of 5.6%, [25]classifying the country as a high-prevalence region for HBV infection and multiple studies have shown regional variations, with the eastern region having a lower carriage rate compared to the central and western regions [26–27]. Blood donors, as a distinct subgroup, reflect the HBV prevalence of the general population and with notable regional differences. For instance, reported HBV disqualification rates among donors vary across cities, such as 0.26% in Beijing [21], 0.68% in Nanjing [28], and 0.75% in Guangzhou [29]. This study reveals an even lower HBV disqualification rate of 0.18%—significantly below the natural population’s carriage rate. This remarkable reduction can be attributed to widespread HBV vaccination, stringent donor screening protocols, and enhanced public health awareness.

Of particular note is the significant elevation in HBsAg positivity to 0.60% during June-July 2021, markedly exceeding rates observed in other periods (Fig 2). Subsequently, the research team conducted an in-depth analysis of the 88 HBsAg (ELISA) single-reagent positive samples during this timeframe. As shown in Table 4, the positivity rate for reagent 1 remained stable at a normal level (0.08%, 7/88), while reagent 2 increased anomalously to 92% (81/88). Nevertheless, all single-positive specimens tested negative for HBV DNA. With consistent testing personnel and regularly maintained/calibrated equipment, operational errors were first excluded from consideration. Upon reevaluation using chemiluminescence, three specimens that tested positive for HBsAb (including one co-positive for HBcAb) were identified from the reagent 2 single-positive group. Multiple validation experiments were performed in collaboration with the manufacturer, confirming that reagent 2, after slight adjustments to its components, produced nonspecific reactions in certain populations, resulting in an increased false-positivity rate. A retrospective analysis revealed that the single-positive population for reagent 2 included 32 repeat successful donors whose historical testing records contradicted recent abnormal results. This discrepancy effectively excludes the possibility of local HBV endemic trend variations. In addition, a nationwide multicenter data comparison indicated this phenomenon was geographically widespread. Technical discussions with the manufacturer confirmed that recent adjustments to the reagent 2 components had occurred, which, despite passing factory validation, led to detection deviations when applied to large samples. Following the research team’s recommendation to optimize the reagent formulation, the HBsAg positivity rate returned to baseline levels in 2023–2024. This case underscores the importance of continuous monitoring and validation of diagnostic reagents to ensure the accurate and reliable surveillance of infectious diseases.

Subgroup analysis of voluntary blood donors (2018–2024) demonstrated significant demographic variations in total reactivity rates (Table 3). The 35–45 age cohort showed the highest reactivity (0.28%, p < 0.001), consistent with global patterns linking middle adulthood to increased exposure risks [30]. Gender disparities mirrored established trends, with male donors exhibiting 1.45-fold higher reactivity compared to female donors (0.45% vs. 0.31%; p < 0.001), which associate male gender with higher-risk behaviors. Notably, first-time donors had nearly double the reactivity rate of repeat donors (0.48% vs. 0.28%), supporting the WHO recommendation for stringent screening of novice donors [31]. The inverse relationship between education level and positivity – with postgraduate donors showing the lowest rate (0.05%) – corroborates Jin Y’s findings [32] on health literacy as a protective factor. These patterns underscore the importance of demographic-specific screening strategies in blood safety protocols.

The evaluation of the donor reentry program (Table 5) shows that from January to July 2021, the reintegration success rate in this region reached 81% (47/58). This demonstrates the substantial effectiveness of the dynamic management system based on verified health records. Current regulations require donors to proactively apply for reintegration 6 months after failed screening, resulting in a relatively low volume of applications. Moreover, the significant financial investment required has limited the implementation of this initiative to a few provinces across the country. The development of a unified national cloud platform for donor health management should be explored. This can leverage information technology to minimize operational costs and facilitate the standardized adoption of donor reintegration mechanisms, which should be the focus of future research.

## Conclusion

Our research group conducted a systematic evaluation of voluntary blood donor screening protocols in East China from 2018 to 2024, with particular attention to regional donor population characteristics. Through implementation of complementary screening modalities (ELISA and NAT testing), we established a robust, multi-layered safety framework that significantly enhanced blood product safety. By conducting a comparative analysis with data from other regions in China, the research group not only demonstrated the effectiveness of the region’s voluntary blood donation strategy in reducing the risk of blood-borne infectious diseases but also highlighted its commitment to safeguarding public health. These findings provide valuable insights for other regions in formulating blood screening policies. Furthermore, the research team pioneered the introduction of a closed-loop management model that integrates a four-dimensional interactive mechanism of confirmation testing, data feedback, social surveys, and donor recall, thereby recovering potentially eligible donors while embodying a humanitarian approach to donor care. Despite the study identifying a slight upward trend in HIV infections, a notable gap exists in traceback research for this specific population. Strengthening pre-donation inquiries and enhancing risk assessment efforts are critical directions that the research team is committed to pursue. These efforts will further refine the screening process and enhance the overall safety and efficacy of blood donation systems.

It is worth noting that the limitations of this study were as follows. First, among NAT-positive/ELISA-negative cases, occult HBV infections (OBI) lacked confirmatory testing and longitudinal monitoring, hindering accurate evaluation of their prevalence and potential transmission risks. Second, A slight increase in HIV positivity was observed in 2024, though it was not statistically significant. The absence of traceback studies or risk behavior assessments in this subgroup limits the ability to develop targeted prevention measures. Future research efforts will prioritize the optimization of pre-donation questionnaires, the refinement of donor risk assessment tools, and the implementation of systematic follow-up protocols

## Supporting information

S1 Data The data underlying the results presented in the study. (XLSX)
